# Controlling the Degree of Functionalization: In‐Depth Quantification and Side‐Product Analysis of Diazonium Chemistry on SWCNTs†

**DOI:** 10.1002/chem.201902330

**Published:** 2019-09-05

**Authors:** Milan Schirowski, Frank Hauke, Andreas Hirsch

**Affiliations:** ^1^ Joint Institute of Advanced Materials and Processes (ZMP) Friedrich-Alexander University of Erlangen-Nürnberg Dr.-Mack-Str. 81 90762 Fürth Germany; ^2^ Chair of Organic Chemistry II Friedrich-Alexander University of Erlangen-Nürnberg Nikolaus-Fiebiger-Str. 10 91054 Erlangen Germany

**Keywords:** diazo compounds, nanotubes, quantification, surfactants, thermogravimetry

## Abstract

We present an in‐depth qualitative and quantitative analysis of a reaction between 4‐iodobenzenediazonium tetrafluoroborate and single‐walled carbon nanotubes (SWCNTs) via thermogravimetric analysis coupled with mass spectrometry (TG‐MS) or a gas chromatography and mass spectrometry (TG‐GC‐MS) as well as Raman spectroscopy. We propose a method for precise determination of the degree of functionalization and quantification of physisorbed aromates, detaching around their boiling point, alongside covalently bonded ones (cleavage over 200 °C). While the presence of some side products like phenol‐ or biphenyl species could be excluded, residual surfactant and minor amounts of benzene could be identified. A concentration‐dependent experiment shows that the degree of functionalization increases with the logarithm of the concentration of applied diazonium salt, which can be exploited to precisely adjust the amount of aryl addends on the nanotube sidewall, up to 1 moiety per 100 carbon atoms.

## Graphical abstract

**Thermogravimetric and Raman** analysis of aryl‐functionalized SWCNTs via diazonium compounds allows for precise quantification of product and side products, even physisorbed ones. Logarithmic concentration‐dependence of the diazonium reactant enables precise adjustment of the degree of functionalization up to 1.0 %.

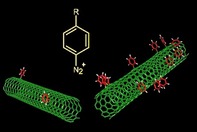

## Introduction

Single‐walled carbon nanotubes (SWCNTs) are nowadays commonly functionalized in order to exploit their unique properties. One of the most prominent functionalization routes is covalent functionalization, which has been applied to improve the nanotubes’ solubility in organic or aqueous solvents,^1^ to separate metallic from semiconducting species,^2^ and to eventually allow for application, for example in supercapacitors,^3^ batteries,^4^ biomedicine,1d, 5 or material reinforcement in polymers.^6^ Among functionalization routes which often require very aggressive chemicals like elemental fluorine,^7^ carbenes, nitrenes,1b, 8 or alkali metals under strictly inert conditions,^9^ one of the rather simple, low‐hazardous, rapid, and non‐destructive pathways is the reaction between neutral SWCNTs and diazonium salts. The reaction is known since 2001 and the underlying single‐electron transfer (SET) mechanism has been studied well.2b, 10 Nevertheless, analytic methods utilized for nanomaterials like SWCNTs are limited mainly due to their polydispersibility, making classic techniques like nuclear magnetic resonance (NMR) and mass spectrometry (MS) unfavorable. Hence, other techniques like Raman‐, absorption‐, and emission spectroscopy, thermogravimetric analysis (TGA), X‐ray photoelectron spectroscopy (XPS), microscopy and rarely infrared (IR) spectroscopy are usually applied to shed light on the chemical identity of the nanomaterial. However, mentioned analytical methods are qualitative or semi‐quantitative at most. A precise analysis of the degree of functionalization or identification of side‐products including the influence of reactant concentration has rarely been performed. Oftentimes, SWCNTs functionalized with the desired functional group is anticipated the only product with mere confirmation by XPS or IR.

In this study, we intended to develop a method to precisely determine the degree of functionalization (DoF) of a selected diazonium salt‐based reaction. Raman spectroscopy, which can be considered standard analysis for many nanomaterials, and forefront analytical tools, consisting of a TGA device coupled with MS (TG‐MS) or a gas chromatograph with MS detector (TG‐GC‐MS) were applied to obtain a complete image of side‐products including aromates deriving from side‐reactions and residual surfactant. Suitable reference experiments were performed to distinguish between non‐covalently and covalently bonded aromates as well as to precisely determine the cleavage temperature of both species. Furthermore, we investigated the influence of diazonium salt concentrations on the reaction, in particular on the degree of functionalization, by applying a large width of reactant concentrations. Moreover, the *I*_D_/*I*_G_ ratio obtained by Raman spectroscopy is commonly used to obtain an impression on whether or not a reaction succeeded yet is known to show down‐turned hyperbolic curve behavior as reported by Cançado et al. for graphene^11^ which precludes simple quantification. For SWCNTs this effect was observed as well but is less thoroughly investigated.10a, 12 Thus, we also analyzed the correlation between the *I*_D_/*I*_G_ ratio and the degree of functionalization in order to elaborate if quantification or at least semi‐quantitative statements based solely on Raman spectroscopy are reasonably feasible.

## Results and Discussion

**Scheme 1:**
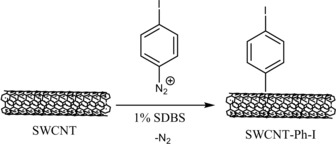
Reaction of SWCNTs with a 4‐iodobenzenediazonium salt in 1 % SDBS solution resulting in the aryl‐functionalized SWCNT‐PhI.

For the present study, nine different concentrations of diazonium salt were added to a SWCNT dispersion in 1 % SDBS (sodium dodecylbenzenesulfonate, which is known to disperse SWCNTs well and results in good spectroscopic features)^13^ solution yielding (4‐iodophenyl)‐functionalized nanotubes (SWCNT‐PhI) (see Scheme 1). Applied concentrations of diazonium salts were within the range of 0.001 mol L^−1^–0.333 mol L^−1^ (for details see Table S1 in the Supporting Information). Lower concentrations were not accomplished, as balancing with sufficient precision isn't facile. At higher concentrations, solubility of the diazonium salt reaches its limit, and stirring turns out to be challenging as the dispersion gets highly viscous. In order to exclude the influence of the reaction procedure (e.g., sonication, solvent and surfactant), a reference sample (Ref. 1a) was treated likewise, but without any diazonium salt.

**Figure 1:**
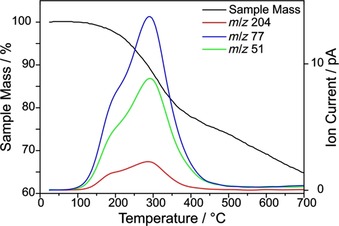
TG‐MS spectrum of SWCNT‐PhI (c(diazonium)=0.083 m). The colored lines indicate the MS traces of the three most prominent iodobenzene masses (*m*/*z* 204 (C_6_H_5_I^⋅+^); *m*/*z* 77 (C_6_H_5_^+^) and *m*/*z* 51 (C_4_H_3_^+^)). The sample mass (TGA spectrum) is depicted as black line.

The obtained functionalized SWCNTs were analyzed by TG‐MS, which is a powerful analytical tool to determine the total mass loss while simultaneously providing information about the chemical identity of the detached molecules. The MS ion current of each functionalized SWCNT‐PhI sample shows the expected mass fragments of iodobenzene (*m*/*z* 204 (C_6_H_5_I^⋅+^); 77 (C_6_H_5_^+^) and 51 (C_4_H_3_^+^)) at temperatures between 150 °C and 400 °C (see Figure 1), which hence will be referred to as “main mass loss region”. While a mass loss is still observable at higher temperatures, it does not derive from the desired sidewall‐bonded aryl moiety, as indicated by the missing MS ion currents. It is likely originating from impurities, residual solvent and surfactant, or lattice decomposition (vide infra).^14^

**Figure 2:**
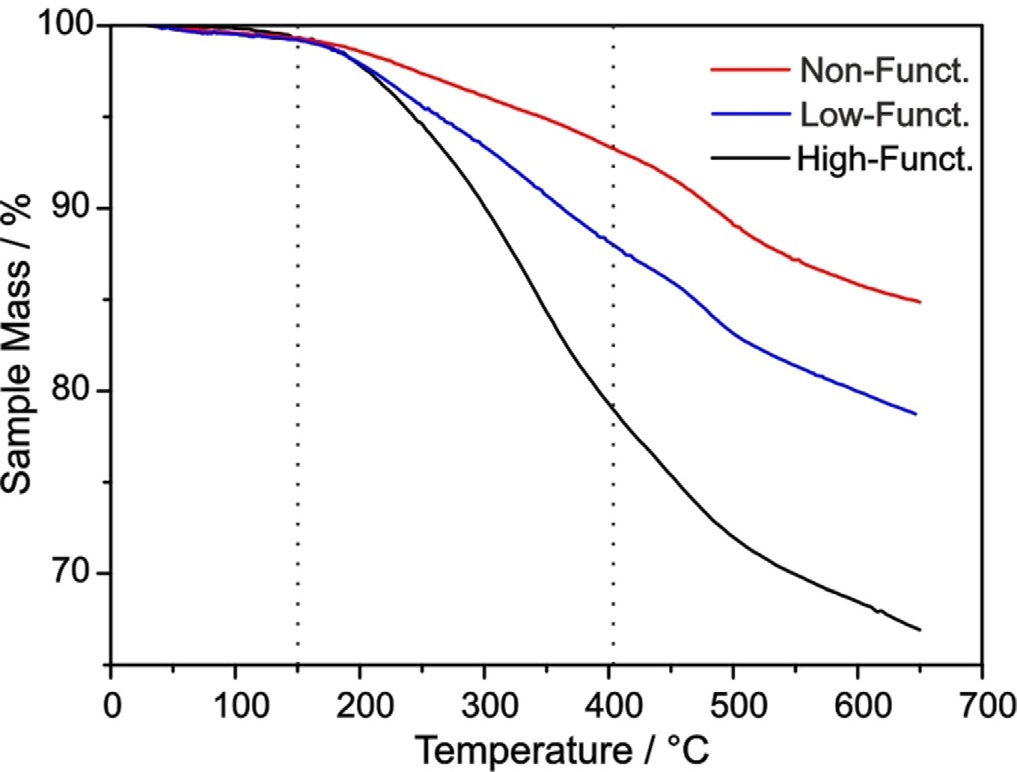
TGA spectra of low‐functionalized (*c*(diazonium)=0.021 m), high‐functionalized (*c*(diazonium)=0.333 m) and non‐functionalized (Ref. 1a) SWCNTs. Dashed lines at 150 °C and 400 °C to highlight the main mass loss region.

However, it is necessary to bear in mind that not 100 % of the mass loss in the main mass loss region originates from the desired iodophenyl moieties. In the reference sample (Ref. 1a), consisting exclusively of SWCNTs sonicated in surfactant solution, a mass loss of 5.9 % can be observed, which might derive from physisorbed water, other non‐covalently bound molecules, or defects which are inherent to the pristine material. Therefore, this considerable value needs to be taken into account in order to calculate an accurate mass loss of iodophenyl groups and thus a degree of functionalization (DoF). As seen in Figure 2, the TGA spectra of three chosen SWCNT‐PhI samples are identical until 150 °C. Then, in the main mass loss region (marked by dashed vertical lines), the difference between high and low functionalization becomes distinct, revealing higher mass losses for higher‐functionalized samples. At even higher temperatures (>400 °C), no indication for a difference in the samples can be found, as the TGA curves are parallel in that region. This corroborates the results of TG‐MS (see Figure 1) and proves that the cleavage of the 4‐iodophenyl group occurs in the main mass loss region between 150 °C and 400 °C. A TG‐MS spectrum of pure SDBS (see Figure S3) shows that the surfactant itself decomposes at around 480 °C, revealing MS ion peaks of *m*/*z* 91 (C_7_H_7_^+^), 77 (C_6_H_5_^+^), and 51 (C_4_H_3_^+^). Since, likewise, both a corresponding mass loss and the corresponding MS peaks (see Figure 2 and Figure 4) appear in the functionalized SWCNT‐PhI samples, one can anticipate that residual surfactant is the source of the underlying mass loss. The SDBS molecule, being physisorbed to the nanotubes, can surpass the washing procedure due to strong van‐der‐Waals interactions with the aromatic ring system of the SWCNTs which is common for molecules that can enclose the nanotubes and/or contain large π‐systems.13a, 15 Alternatively, the high mass loss temperature and the toluene peak are an indicator for lattice decomposition, which has been previously reported for graphene^14^ and SWCNTs.^16^ Eventually, the mass loss deriving exclusively from the desired functional group Δ*m*(FG) can be calculated using Equation (1):

(1) $$\Delta m\left(FG\right)={\Delta m}_{400-150}-{\Delta m}_{Ref,400-150}$$

where Δ*m*_400−150_ is the mass loss of the sample in the main mass loss region and Δ*m*_Ref,400−150_ is the mass loss of the reference sample (Ref. 1a) in that temperature range. Subsequently, the DoF can be calculated utilizing Equation (2):

(2) $$DoF=\frac{\Delta m\left(FG\right){}^{*}M\left(C\right)}{m\left(C\right){}^{*}M\left(FG\right)}$$

where DoF is the degree of functionalization, *M*(C) the molar mass of carbon (12.01 g mol^−1^), *m*(C) the mass of carbon in the nanotube lattice (remaining sample mass at 650 °C) and *M*(FG) the molar mass of the functional group (203.00 g mol^−1^ for iodophenyl).

**Figure 3:**
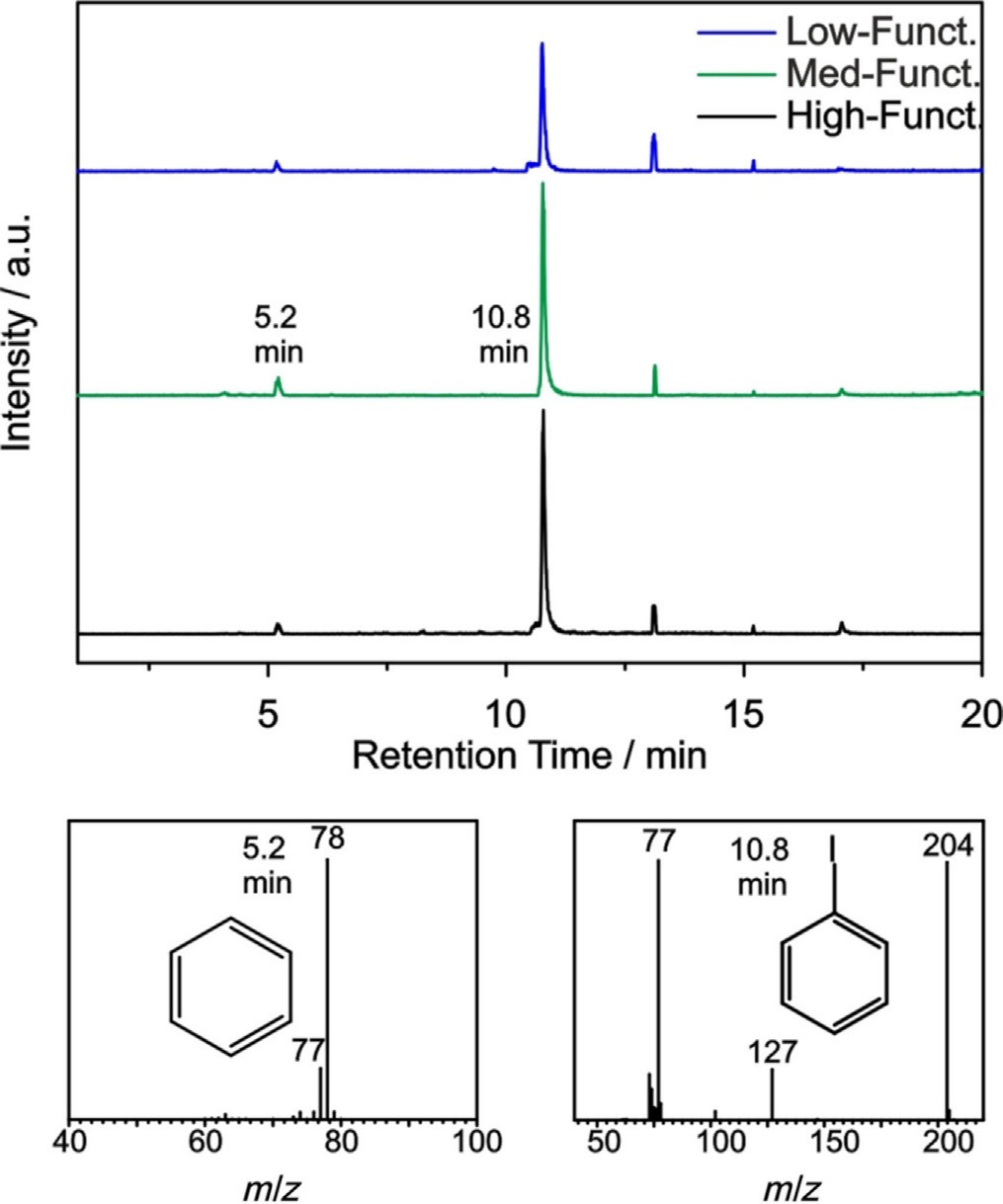
TG‐GC‐MS spectra. Top: total ion chromatogram (TIC) of three SWCNT‐PhI samples with low (blue, *c*(diazonium)=0.003 m), medium (green, *c*(diazonium)=0.021 m) and high (black, *c*(diazonium)=0.333 m) degree of functionalization. Bottom: MS spectra of benzene (*t*_R_=5.2 min) and iodobenzene (*t*_R_=10.8 min).

Despite being a powerful tool to reveal information about volatile compounds created during the heating of the sample, TG‐MS is a limited analytical method, especially when multiple molecules are cleaved from the sample and detected simultaneously causing an overlap of the MS signals. However, this drawback can be avoided when a chromatographic step is inserted between the heating (i.e., TGA) and detection (i.e., MS) step, allowing to distinguish multiple volatile compounds.^17^ Thus, a TG‐GC‐MS system was applied to unambiguously identify the molecules formed in the gas phase of the SWCNT‐PhI sample. In our case, the gas phase at 280 °C was injected into the GC‐MS system, where iodobenzene and, to a much lower extent, benzene, could be identified as only molecules present in the gas phase (see Figure 3). The injection was set to 280 °C by reason that the highest mass loss and the highest MS ion current were observed around this temperature (compare Figure 1). Benzene, with a retention time (*t*_R_) of 5.2 min, most likely derives from phenyl groups on the SWCNT sidewall as a result of side reactions. However, the marginal amount of this side product is difficult to quantify but can be neglected compared to the amount of iodobenzene detected at *t*_R_=10.8 min. To avoid phenyl groups as a side product, diazonium salts with more redox‐stable moieties, like 4‐*tert*‐butylbenzenediazonium, could be used in future works. However, the chosen 4‐iodophenyl group can be of great interest as it allows for further coupling via Sonogashira or Ullmann type reactions.^18^ GC peaks at retention times higher than 10.8 min are revealed in the chromatogram as well, but can likewise be detected in reference runs and hence do not derive from the SWCNT samples. By using MS, those peaks can be identified as siloxanes and therefore attributed to fragments from the GC column (so‐called “column bleeding”).

Having proven by TG‐GC‐MS that iodobenzene, alongside negligible amounts of benzene, is the only molecule cleaved from the SWCNT‐PhI sample in the main mass loss region, the question arises whether it is covalently bonded or only physisorbed onto the SWCNTs. Temperature‐dependent Raman spectroscopy has been utilized in several publications to prove that a rehybridization from sp^3^ carbon atoms to sp^2^ (rearomatization) takes place, proving the covalent attachment of the desired aryl moieties as well as the reversibility of the reaction.^14^, ^19^ However, physisorbed aromates alongside the C−C bonded aryl moieties could not be excluded. Utilizing a simple reference experiment in the present study, physically mixing iodobenzene with a SWCNT dispersion, allows for confirmation of the coexistence of physisorbed iodobenzene and moreover quantification of both species.

**Figure 4:**
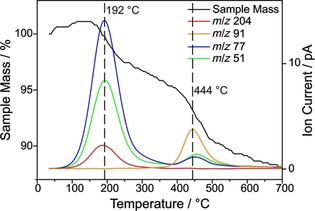
TG‐MS spectrum of the physical mixture of SWCNTs with iodobenzene (Ref. 1b). Colored lines indicate the three most prominent masses of iodobenzene (*m*/*z* 204 (C_6_H_5_I^⋅+^); *m*/*z* 77 (C_6_H_5_^+^) and *m*/*z* 51 (C_4_H_3_^+^)) and of toluene (*m*/*z* 51, *m*/*z* 77, *m*/*z* 91 (C_7_H_7_^+^)). The sample mass (TGA spectrum) is depicted as black line. Dashed vertical lines with indicated temperatures at the MS peaks point out the cleavage temperatures.

Figure 4 shows the TG‐MS spectrum of SWCNT samples with non‐covalently bound iodobenzene, applying the same procedure as for the covalent approach (see Scheme 1), utilizing iodobenzene instead of the 4‐iodobenzendiazonium salt (Ref. 1b). The highest mass loss and MS ion current appear at a lower temperature (191 °C) than for the covalent SWCNT‐PhI samples. This temperature neatly fits to the boiling point of iodobenzene (188 °C), which hints that only physisorption rather than covalent bonding took place. This assumption can be corroborated by Raman spectroscopy showing that *I*_D_/*I*_G_ ratios of Ref. 1b are unchanged compared to pristine material (*I*_D_/*I*_G_^532^=0.10, *I*_D_/*I*_G_^633^=0.06, *I*_D_/*I*_G_^785^=0.11, Figure S4), and further by the fact that no reasonable activation step was applied which could potentially lead to the formation of C−C‐bonds under the chosen conditions. Utilizing Equation (1) and Equation (2), the amount of non‐covalently bound iodobenzene on the SWCNT surface can be calculated to account for ca. 0.13 % in Ref. 1b. Considering that aryldiazonium salts in aqueous dispersion decompose to the corresponding arene and phenol^20^ it is plausible that a certain amount of iodobenzene is formed as a side product during the covalent approach and thus gets physisorbed to the SWCNTs. Next to iodobenzene, other molecules like 4‐iodophenol or biphenyl species like 4,4′‐dioodobiphenyl can be expected to be formed as decomposition product of the diazonium salt and thus get physisorbed by the SWCNT surface as well. However, TG‐GC‐MS proved their absence in all samples (see Figure 3). This can be explained by an enhancement of water solubility for the phenol, and by a statistically very unlikely radical recombination of two iodophenyl radicals.

**Figure 5:**
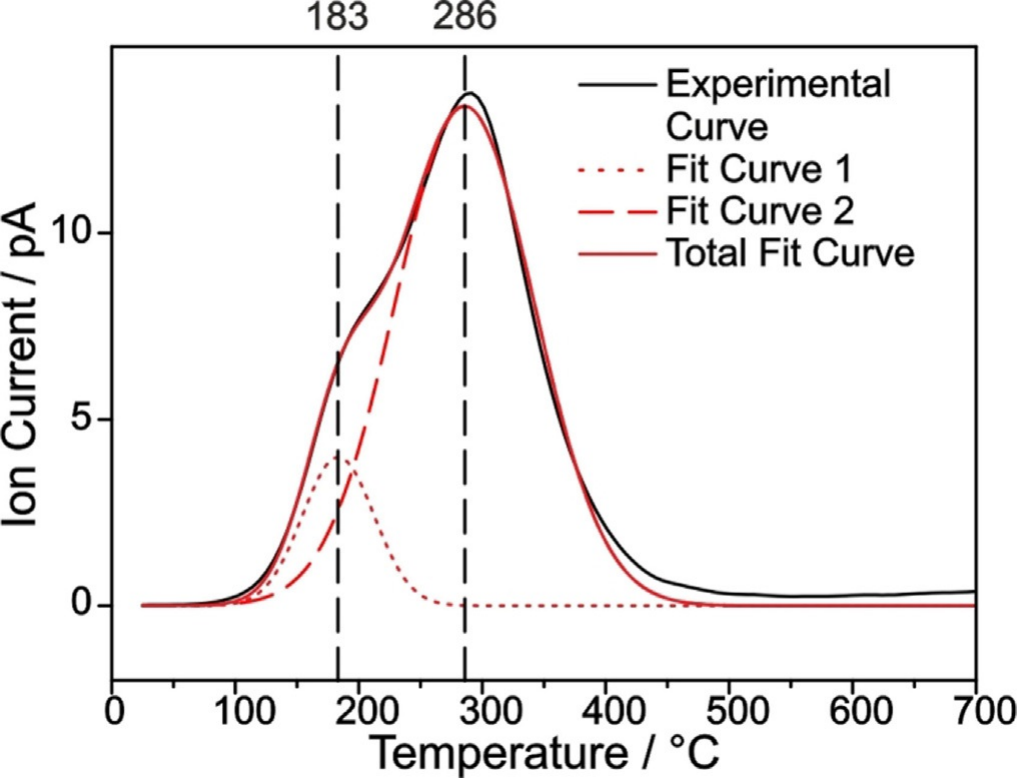
Experimental MS curve from the TG‐MS experiment (*m*/*z* 77, black line) of functionalized SWCNT‐PhI (c(diazonium)=0.083 mol L^−1^) with corresponding fit curve (red line) consisting of two single Gaussian curves (dashed red lines). Black dashed vertical lines point out the cleavage temperatures of the covalently and non‐covalently bound aryl moieties.

Having a closer look at the TG‐MS spectrum of the diazonium approach (see Figure 1), one can see a shoulder of the iodobenzene peaks at ca. 190 °C which we can now, thanks to Ref. 1b, attribute to physisorbed iodobenzene. Utilizing a suitable fitting function two Gaussian curves can be fit into the MS signals (see Figure 5, here *m*/*z* 77 was used because it was the most intense signal). One Gaussian fit curve has its peak at a temperature of 183 °C, matching the cleavage temperature of the non‐covalent species (see Figure 4) as well as the boiling point of iodobenzene. The peak of the other Gaussian fit curve, which by exclusion can now be attributed to purely covalently bonded 4‐iodophenyl groups, is located at 286 °C. A Lorentzian fit curve, which was also taken into consideration because it is usually applied for spectroscopic features like Raman signals,^21^ did not provide a satisfactory fit curve. The area of the MS peaks allows for the quantification of the covalent and non‐covalent amount of iodobenzene, revealing that roughly 87 % of the iodobenzene is bonded covalently to the SWCNTs sidewall and 13 % non‐covalently.

**Figure 6:**
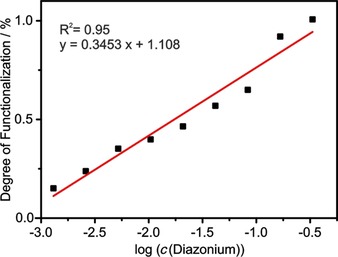
Degree of functionalization of nine functionalized SWCNT‐PhI samples as calculated by Equations (1) and (2) and plotted as a function of log(*c*(diazonium)) (based on mol L^−1^, black squares). Red line: fitting linear slope with indicated equation and stability index R^2^.

To shed light on the influence on the degree of functionalization, nine different concentrations of diazonium reactant were applied for the synthesis of SWCNT‐PhI. One might expect a linear increase of the grafting ratio with rising diazonium concentrations up to a point where no free space on the SWCNT sidewall is available for further aryl moieties, resulting into an anticipated saturation curve. However, the results reveal a logarithmic correlation when plotting the calculated degree of functionalization [as determined by Eq. (1) and Eq. (2)] against the concentration of applied diazonium salt (see Figure 6). For classic analytical chemistry correlation coefficients of *R* ^2^ >0.99 are demanded for a satisfactory linear slope. However, the obtained *R* ^2^ values higher than 0.90 are excellent considering the quantification of functional addends on nanomaterials. Note that the amount of phenyl groups as side product and the amount of physisorbed iodobenzene were ignored for this calculation, as including them would require a TG‐MS evaluation for each single sample. Further, it should be mentioned that the highest yielded degrees of functionalization of ca. 1 % (e.g., 1 in 100 carbon atoms carries an aryl group) are far lower than the theoretical limit, as already the sample with the lowest diazonium concentration contains one diazonium cation for 64 nanotube carbon atoms. Analogous to the reported C_60_Br_24_ revealing a 1,4‐addition pattern, likewise conceivable for aromates on SWCNT sidewalls, the grafting ratio could potentially increase up to 40 %.^22^ Hence, only a minor fraction of applied diazonium salt molecules eventually covalently bond to the SWCNT sidewalls at high concentrations. Therefore, side reactions like the decomposition of the diazonium cation are predominant.

**Figure 7:**
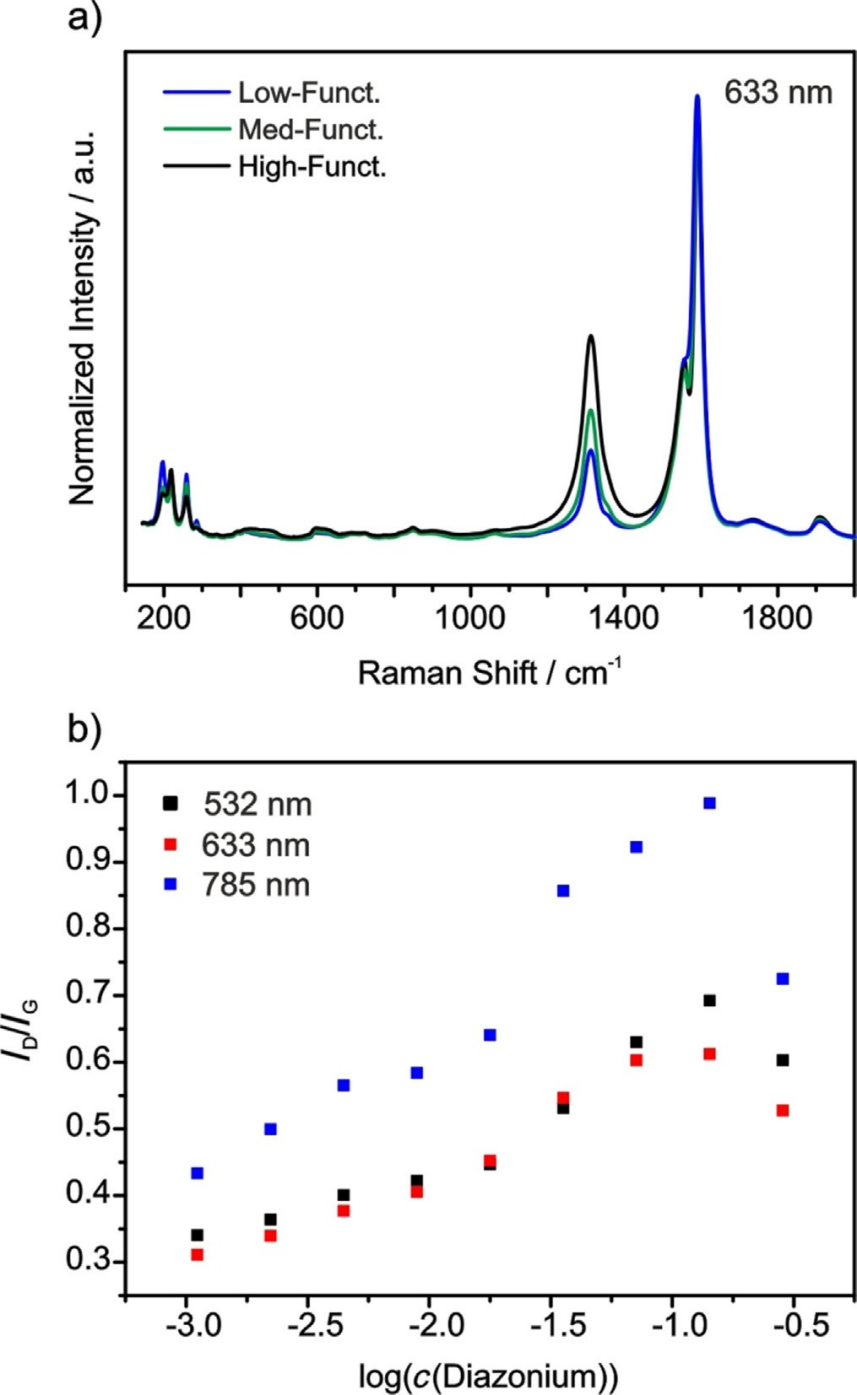
a) Raman mean spectra of low‐functionalized (*c*(diazonium)=0.0013 m), medium‐functionalized (*c*(diazonium)=0.010 m) and high‐functionalized (*c*(diazonium)=0.083 m) SWCNT‐PhI samples with an excitation wavelength of 633 nm. Spectra normalized to the G‐band. b) *I*_D_/*I*_G_ ratios of nine SWCNT‐PhI samples with varying concentrations of applied diazonium salt at 532 nm (black), 633 nm (red) and 785 nm (blue) excitation wavelength.

**Figure 8:**
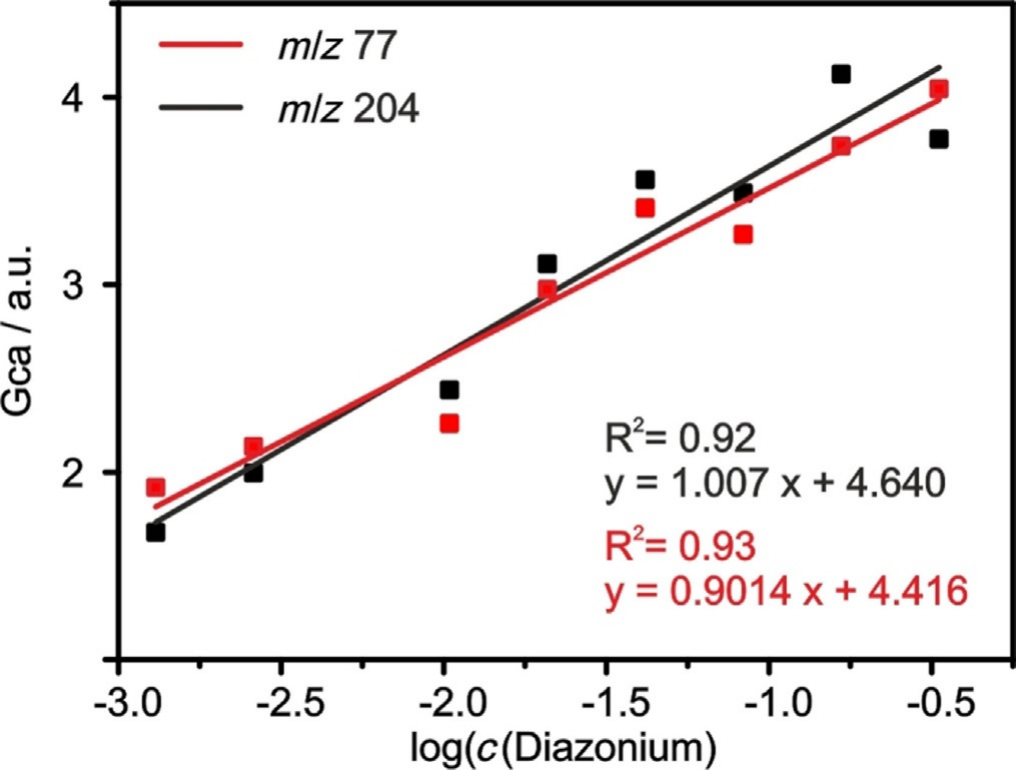
Experimental GC areas of the ion tracks *m*/*z* 77 (C_6_H_5_^+^, red line) and *m*/*z* 204 (C_6_H_5_I^⋅+^, black line) in dependence of applied diazonium salt concentrations. GCa is the specific GC area, meaning GC area normalized per mg sample.

Utilizing Raman spectroscopy, the *I*_D_/*I*_G_ ratio is known to increase with increasing defect density. Yet, Cançado et al. have shown that the correlation is not linear in the case of graphene, as the *I*_D_/*I*_G_ ratios drops at very high amounts of defects.^11^ A similar behavior was observed for SWCNTs,10a, 12 but up to date not investigated as thoroughly as for graphene. For nanotubes, the *I*_D_/*I*_G_ ratio also highly depends on the laser excitation energy,^19^ which further complicates a precise quantification of defect density. To investigate the influence of the degree of functionalization on the Raman spectroscopic properties, statistical Raman spectroscopy (SRS) was performed with >2000 single point spectra for each sample and three different laser excitation wavelengths (532, 633, and 785 nm, see Figure 7). The 785 nm laser yields the highest *I*_D_/*I*_G_ ratios. However, SWCNTs with different chiralities, metallicities, and diameters are probed by varying excitation lasers, leaving this absolute value rather insignificant.^23^ Despite, independently of the utilized excitation wavelength, the highest *I*_D_/*I*_G_ ratio is observed at diazonium concentrations of 0.16 m corresponding to a degree of functionalization of roughly 0.9 %. At higher concentrations of applied diazonium salt, a drop of the *I*_D_/*I*_G_ ratio is observed. Another potential explanation for this phenomenon, aside from the hyperbolic behavior researched by Cançado, would be that excessive amounts of diazonium salt in fact lead to poorer degrees of functionalization. However, this can be disproven by TGA (see Figure 6) and GC‐MS results (see Figure 8, vide infra), both clearly demonstrating an increase of the degree of functionalization upon higher diazonium concentrations.

To further corroborate the fact that indeed a logarithmic correlation between the concentration of the diazonium salt and the degree of functionalization is underlying, the areas of the TG‐GC‐MS spectra were investigated. Hof et al. have shown that the amount of functional groups cleaved off SWCNTs is directly proportional to the areas in the gas chromatograms.^17^

Figure 8 shows that the peak areas of the gas chromatograms deriving from the TG‐GC‐MS experiments correlate logarithmically with the applied concentration of diazonium salt, just like the TGA mass loss (see Figure 6). This proves that the higher mass released from the SWCNT‐PhI samples indeed derives from higher amounts of aryl functionalities on the nanotube sidewall rather than from increasing amounts of side products. Thus, it confirms the logarithmic correlation between the concentration of applied diazonium salt and the DoF. Furthermore, the result verifies that Raman spectroscopy shows a non‐linear “Cançado‐like” behavior making it an unsatisfactory tool for precise quantification, in particular when the degree of functionalization exceeds 0.9 %.

As a final confirmation that the increasing mass loss indeed derives from higher amounts of aryl groups covalently bonded to the SWCNT sidewall, suitable reference experiments were performed (Ref. 2 and Ref. 3, see Table S1). They reveal that the addition of iodobenzene (see Figure S1) or biphenyl species (see Figure S2) lead to no significant difference in the TGA spectra of the samples. Hence, relevant amounts of the mentioned aromates are not physisorbed to the SWCNTs, and the higher mass loss derives exclusively from higher quantities of covalently bonded aromates.

The obtained results allow for an interesting novelty in tailor‐made functionalization of SWCNT. For the first time, a desired degree of functionalization can be adjusted, at least in the range of 0.2–1.0 %. For this purpose, three different concentrations of diazonium salts need to be reacted with a nanotube dispersion. Subsequently, the resulting functionalized SWCNT‐PhI should be analyzed by TG‐MS and the degree of functionalization (DoF) calculated via Equation (1) and (2). From the obtained regression curve in the semi‐logarithmic plot (see Figure 6), the necessary concentration of diazonium salt required for the desired degree of functionalization can be interpolated. By the analytical techniques described above, the purity of the obtained material as well as the amount of physisorbed aromates can be determined. Alternatively, a calibration utilizing Raman spectroscopy can employ for a very quick, but equally raw estimation of the DoF. When changing the type of diazonium salt (varying in reactivity due to electron‐withdrawing or ‐donating moieties)2b or the type of SWCNT starting material (varying in reactivity due to diameter, metallicity, inherent defects, and other factors), mentioned calibration procedure will most likely require modification. Whether this method can be adapted for the investigation of other nanomaterials such as graphene, MWCNTs, or non‐carbon‐based ones needs to be investigated in future works.

**Figure 9:**
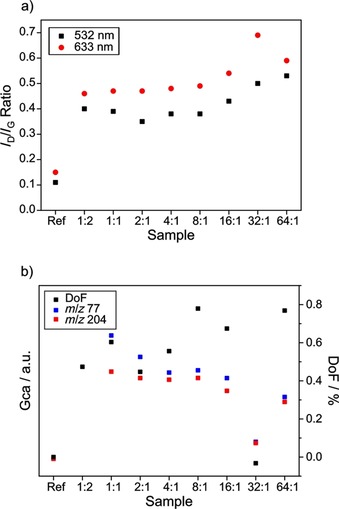
Raman, TGA and GC‐MS data of the excess diazonium samples. a) *I*_D_/*I*_G_ ratios obtained by SRS with 532 and 633 nm laser excitation wavelength. b) Specific GC areas (GCa) of *m*/*z* 77 (blue) and 204 (red), representative of iodobenzene. Degree of functionalization (DoF) as calculated from TGA data by Equation (1) and (2) depicted in black. GCa normalized per mg sample. GC area of sample 1:2 not recorded due to machine failure.

Lastly, we desired to further enhance our understanding of the underlying reaction. It was our aim to shed light on whether it is possible to increase the DoF unrestrictedly by tremendously increasing the amount of added diazonium salt, or if, eventually, a threshold of sidewall functionalities will be reached upon thermodynamic saturation. As mentioned earlier, however, higher concentrations of diazonium salts were challenging to accomplish in the reaction mixture under the chosen conditions. Hence, another experimental series was conducted, highly diluting the dispersion of SWCNTs in aqueous surfactant (experimental details see Table S2). This way, it was possible to expose the nanotube to a vast excess of diazonium salt up to a ratio of 64:1 (*n*(diazonium) : *n*(carbon)). In contrast, the first series was limited to a ratio of 4:1. Figure 9 a depicts the Raman spectroscopic data of the excess diazonium experiment. The *I*_D_/*I*_G_ ratios at both probed laser excitation wavelengths (532 and 633 nm) are more or less constant for all present samples except for the samples with the highest diazonium salt excess. In the latter two samples (32:1 and 64:1), the *I*_D_/*I*_G_ values are significantly increased, indicating a lower (!) degree of functionalization, considering being on the high‐concentration side of the hyperbolic Cançado curve (compare Figure 7 a).^11^ For sample 16:1, a slight increase can be observed as well.

To corroborate the Raman data, TG‐GC‐MS experiments were performed and summarized in Figure 9 b, depicting the specific GC area of the most prominent iodobenzene masses (*m*/*z* 77 and 204) alongside the calculated DoF [see Eq. (1) and (2)]. No clear trend is observable considering the diazonium concentration and the corresponding DoF. Rather, the calculated amount of grafted aryl addends seems to intensely fluctuate, which was not the case for the first experimental series (compare Figure 6). This is most likely due to an insufficient precision of the TGA balance in relation to the small amounts of samples. In the present experiment, highly diluted SWCNT samples had to be utilized in order to allow for the required high diazonium‐to‐carbon ratios, often yielding less than 1 mg of functionalized nanotube sample. While this amount is too small for most conventional TGA machines, it is sufficient for MS and Raman experiments. Therefore, the specific GC areas elucidate the matter, providing more stable and reliable information about the samples. Both probed GCa values are predominantly constant for samples 1:1, 2:1, 4:1, and 8:1, corroborating the previous Raman results and indicating a stable degree of functionalization. Merely the *m*/*z* 77 area is slightly increased for sample 1:1, which can be anticipated a statistical outlier. On the other hand, the three samples with the highest diazonium excess reveal a drop (distinct for 64:1 and 32:1, less pronounced for 16:1) of the GCa, indicating a lower amount of covalent 4‐iodophenyl moieties. The TGA spectra of those high‐excess samples (see Figure S3) reveal an additional mass loss step at 630 °C, which is not observable for SWCNT samples treated with lower amounts of diazonium salt. The origin of this mass loss is unclear, but it is definitely too high to derive from covalent aryl moieties. Much more likely, it seems to be caused by side reactions like polymerization of the diazonium reactant or a damaging of the nanotubes’ lattice.^17^

Summarizing, the experiment with high diazonium excess has illustrated the restrictions of the examined reaction between neutral SWCNTs and diazonium salts. Raman spectroscopy and TG‐GC‐MS analysis have uniformly displayed that the degree of functionalization (DoF) cannot be increased indefinitely by addition of unconfined amounts of diazonium reactant. Rather, saturation is reached at DoFs of around 1 %, and addition of tremendous amounts of the highly reactive diazonium compound results in a descent of aryl moieties accompanied by undesired side reactions.

## Conclusions

We have thoroughly investigated the reaction between nanotubes and diazonium salts by the basis of a model system consisting of 4‐iodobenzenediazonium tetrafluoroborate and HiPco SWCNTs. We found that the 4‐iodophenyl groups covalently bonded to the SWCNTs as main product get cleaved off the SWCNTs in the main mass loss region between 150 °C and 400 °C. Negligible amounts of benzene could be detected via TG‐GC‐MS, indicating partial removal of the iodine. For the first time we were able to prove and quantify physisorbed iodobenzene species in the functionalized samples, which detaches from the nanotubes around its boiling point of 188 °C, alongside the covalently bonded aryl group. In contrast, covalent C−C‐bonds of the aryl‐SWCNT junctions are broken at 286 °C. Applying varying concentrations of diazonium salt onto a constant amount of SWCNTs revealed a logarithmic correlation between the degree of functionalization (DoF) and diazonium concentration as corroborated by TGA mass loss and GC‐MS area analysis. This allows for a precise adjustment of the degree of functionalization in future works after brief calibration. The maximum possible DoF utilizing this reaction is around 1 %. Tremendously increasing the diazonium reactant concentration (*n*(diazonium):*n*(carbon) >8:1) results in an opposed effect, decreasing the amount of 4‐iodophenyl moieties on the SWCNT sidewall and promoting side reactions. Moreover, when Raman spectroscopy was conducted, a hyperbolic “Cançado‐like” behavior for *I*_D_/*I*_G_ ratios was observed, with maximum *I*_D_/*I*_G_ values at a degree of functionalization of 0.9 %, decreasing at higher DoFs. This establishes Raman spectroscopy as a decent semi‐quantitative method for low grafting ratios (e.g., <0.9 %), but futile if used exclusively and precise quantification is necessary.

## Experimental Section

### Used materials

HiPco SWCNTs (diameter 0.8–1.2 nm, length 100–1000 nm) were purchased by Unidym (batch #P2772). All other chemicals were purchased by Sigma–Aldrich and used without further treatment if not stated otherwise.

### Synthesis of 4‐iodobenzenediazonium tetrafluoroborate

Modified from a known procedure.^24^ 63 mmol of 4‐iodoaniline was dispersed in 250 mL of water. Then, 25 mL HBF_4_ solution (47 %) was added forming a neat purple solution. The solution was cooled with an ice bath and 116 mmol of saturated NaNO_2_ solution was added dropwise via dropping funnel, not exceeding 5 °C. After complete addition, the formed yellowish precipitate was collected, filtered, and washed with 50 mL of ice‐cold water and 50 mL diethyl ether. After finally drying in vacuo for 24 h, the diazonium salt was yielded (78 %).

### Synthesis of (4‐iodophenyl)‐SWCNT

Reaction equation in Scheme 1. 6.0 mg (0.50 mmol) of HiPco SWCNTs was dispersed in 6.0 mL of 1 % SDBS‐solution by sonicating for 30 min. The black dispersion was then cooled to room temperature and 4‐iodobenzenediazonium tetrafluoroborate added at once. Initial applied concentration of the diazonium salt was 0.333 mol L^−1^ (636 mg). For the series, the diazonium concentration was diluted by factor 2 until reaching 1.3 mmol L^−1^ (2.5 mg), corresponding to molar ratios between 4:1 and 1:64 (*n*(diazonium) : *n*(carbon)). The dispersion was stirred for 24 h and subsequently filtered through a 0.25 μm cellulose membrane and washed with 100 mL of water and acetone each. The black solid was scraped off the buckypaper and dried in vacuo for 24 h. Details see Table S1.

For the excess diazonium samples, a similar reaction procedure was applied, utilizing 3.0 mg (0.25 mmol) of SWCNTs dispersed in 500 mL of SDBS solution instead. This allowed for addition of higher amounts of diazonium salt, ranging from 39.7 mg to 5.1 g, corresponding to molar ratios of 1:2–64:1 (*n*(diazonium):*n*(carbon)). Details see Table S2.

### Synthesis of reference samples

The same procedure as for (4‐iodophenyl)‐SWCNT was applied (sonication, stirring, filtering and washing), adding to the reactive diazonium compound iodobenzene, biphenyl, 4,4′‐diiodobiphenyl, or no reactant at all for the non‐functionalized sample. Details see Table S1.

### Raman spectroscopy

Measurements were performed on a LabRam Aramis machine. Maps of 100×100 μm were recorded with a step size of 2 μm, giving a total of 2601 single‐point spectra with acquisition times of 0.1 s. Utilized laser excitation wavelengths were 532 nm, 633 nm and 785 nm, laser power was <1 W to avoid defunctionalization.

### TGA measurements

TG‐MS (online) was performed on a Netzsch Skimmer STA 409 CD. TG‐GC‐MS for performed on a Pyris 1 TGA from PerkinElmer coupled with a Clarus 680 gas chromatograph with Elite‐5MS column and an SQ 8 quadrupole mass spectrometer. Samples were heated with a constant rate of 20 K min^−1^ from room temperature to 700 °C.

## Conflict of interest

The authors declare no conflict of interest.

## Supporting information

As a service to our authors and readers, this journal provides supporting information supplied by the authors. Such materials are peer reviewed and may be re‐organized for online delivery, but are not copy‐edited or typeset. Technical support issues arising from supporting information (other than missing files) should be addressed to the authors.

Supplementary

Supplementary

## References

[1a] K. Kelly , I. Chiang , E. Mickelson , R. Hauge , J. Margrave , X. Wang , G. Scuseria , C. Radloff , N. Halas , Chem. Phys. Lett. 1999, 313, 445–450;

[1b] M. Holzinger , O. Vostrowsky , A. Hirsch , F. Hennrich , M. Kappes , R. Weiss , F. Jellen , Angew. Chem. Int. Ed. 2001, 40, 4002–4005; 12404474

[1c] V. Georgakilas , K. Kordatos , M. Prato , D. M. Guldi , M. Holzinger , A. Hirsch , J. Am. Chem. Soc. 2002, 124, 760–761; 11817945 10.1021/ja016954m

[1d] Z. Liu , X. Sun , N. Nakayama-Ratchford , H. Dai , ACS Nano 2007, 1, 50–56; 19203129 10.1021/nn700040t

[1e] C. A. Dyke , J. M. Tour , J. Phys. Chem. A 2004, 108, 11151–11159.

[2a] B. Gebhardt , F. Hof , C. Backes , M. Müller , T. Plocke , J. Maultzsch , C. Thomsen , F. Hauke , A. Hirsch , J. Am. Chem. Soc. 2011, 133, 19459–19473; 22035086 10.1021/ja206818n

[2b] Y.-J. Do , J.-H. Lee , H. Choi , J.-H. Han , C.-H. Chung , M.-G. Jeong , M. S. Strano , W.-J. Kim , Chem. Mater. 2012, 24, 4146–4151;

[2c] W.-J. Kim , N. Nair , C. Y. Lee , M. S. Strano , J. Phys. Chem. C 2008, 112, 7326–7331;

[2d] W.-J. Kim , M. L. Usrey , M. S. Strano , Chem. Mater. 2007, 19, 1571–1576.

[3] M. De Marco , F. Markoulidis , R. Menzel , S. M. Bawaked , M. Mokhtar , S. A. Al-Thabaiti , S. N. Basahel , M. S. Shaffer , J. Mater. Chem. A 2016, 4, 5385–5389.

[4] S. Zeng , H. Chen , H. Wang , X. Tong , M. Chen , J. Di , Q. Li , Small 2017, 13, 1700518. 10.1002/smll.201700518 28594437

[5] A. Bianco , K. Kostarelos , M. Prato , Curr. Opin. Chem. Biol. 2005, 9, 674–679. 16233988 10.1016/j.cbpa.2005.10.005

[6a] C. A. Mitchell , J. L. Bahr , S. Arepalli , J. M. Tour , R. Krishnamoorti , Macromolecules 2002, 35, 8825–8830;

[6b] J. N. Coleman , U. Khan , Y. K. Gun′ko , Adv. Mater. 2006, 18, 689–706;

[6c] O. Breuer , U. Sundararaj , Polym. Compos. 2004, 25, 630–645;

[6d] S. Ahir , Y. Huang , E. Terentjev , Polymer 2008, 49, 3841–3854.

[7a] K. Bushimata , S.-I. Ogino , K. Hirano , T. Yabune , K. Sato , T. Itoh , K. Motomiya , K. Yokoyama , D. Mabuchi , H. Nishizaka , J. Phys. Chem. C 2014, 118, 14948–14956;

[7b] E. Mickelson , C. Huffman , A. Rinzler , R. Smalley , R. Hauge , J. Margrave , Chem. Phys. Lett. 1998, 296, 188–194.

[8a] M. Holzinger , J. Abraham , P. Whelan , R. Graupner , L. Ley , F. Hennrich , M. Kappes , A. Hirsch , J. Am. Chem. Soc. 2003, 125, 8566–8580; 12848565 10.1021/ja029931w

[8b] C. Gao , H. He , L. Zhou , X. Zheng , Y. Zhang , Chem. Mater. 2009, 21, 360–370.

[9a] J. Gebhardt , S. Bosch , F. Hof , F. Hauke , A. Hirsch , A. Görling , J. Mater. Chem. C 2017, 5, 3937–3947;

[9b] F. Hof , S. Bosch , S. Eigler , F. Hauke , A. Hirsch , J. Am. Chem. Soc. 2013, 135, 18385–18395; 24256165 10.1021/ja4063713

[9c] D. Voiry , O. Roubeau , A. Pénicaud , J. Mater. Chem. 2010, 20, 4385–4391;

[9d] F. Liang , J. M. Beach , K. Kobashi , A. K. Sadana , Y. I. Vega-Cantu , J. M. Tour , W. Billups , Chem. Mater. 2006, 18, 4764–4767;

[9e] F. Liang , A. K. Sadana , A. Peera , J. Chattopadhyay , Z. Gu , R. H. Hauge , W. Billups , Nano Lett. 2004, 4, 1257–1260.

[10a] M. S. Strano , C. A. Dyke , M. L. Usrey , P. W. Barone , M. J. Allen , H. Shan , C. Kittrell , R. H. Hauge , J. M. Tour , R. E. Smalley , Science 2003, 301, 1519–1522; 12970561 10.1126/science.1087691

[10b] J. L. Bahr , J. M. Tour , Chem. Mater. 2001, 13, 3823–3824;

[10c] J. L. Bahr , J. M. Tour , J. Mater. Chem. 2002, 12, 1952–1958;

[10d] G. Schmidt , S. Gallon , S. Esnouf , J. P. Bourgoin , P. Chenevier , Chem. Eur. J. 2009, 15, 2101–2110; 19142944 10.1002/chem.200801801

[10e] C. A. Dyke , M. P. Stewart , F. Maya , J. M. Tour , Synlett 2004, 155–160.

[11] L. G. Cançado , A. Jorio , E. M. Ferreira , F. Stavale , C. Achete , R. Capaz , M. Moutinho , A. Lombardo , T. Kulmala , A. Ferrari , Nano Lett. 2011, 11, 3190–3196. 21696186 10.1021/nl201432g

[12] R. Graupner , J. Raman Spectrosc. 2007, 38, 673–683.

[13a] V. C. Moore , M. S. Strano , E. H. Haroz , R. H. Hauge , R. E. Smalley , J. Schmidt , Y. Talmon , Nano Lett. 2003, 3, 1379–1382;

[13b] M. Islam , E. Rojas , D. Bergey , A. Johnson , A. Yodh , Nano Lett. 2003, 3, 269–273.

[14] G. Abellán , M. Schirowski , K. F. Edelthalhammer , M. Fickert , K. Werbach , H. Peterlik , F. Hauke , A. Hirsch , J. Am. Chem. Soc. 2017, 139, 5175–5182. 28322052 10.1021/jacs.7b00704

[15a] N. Nakashima , Sci. Technol. Adv. Mater. 2006, 7, 609–616;

[15b] D. Tasis , N. Tagmatarchis , V. Georgakilas , M. Prato , Chem. Eur. J. 2003, 9, 4000–4008; 12953186 10.1002/chem.200304800

[15c] M. Zheng , A. Jagota , E. D. Semke , B. A. Diner , R. S. Mclean , S. R. Lustig , R. E. Richardson , N. G. Tassi , Nat. Mater. 2003, 2, 338–342. 12692536 10.1038/nmat877

[16] M. Schirowski , G. Abellán , E. Nuin , J. Pampel , C. Dolle , V. Wedler , T.-P. Fellinger , E. Spiecker , F. Hauke , A. Hirsch , J. Am. Chem. Soc. 2018, 140, 3352–3360. 29405064 10.1021/jacs.7b12910

[17] F. Hof , R. A. Schäfer , C. Weiss , F. Hauke , A. Hirsch , Chem. Eur. J. 2014, 20, 16644–16651. 25345583 10.1002/chem.201404662

[18a] R. Kumar , C. Rao , J. Mater. Chem. A 2015, 3, 6747–6750;

[18b] A. S. Jombert , M. K. Bayazit , C. R. Herron , K. S. Coleman , D. A. Zeze , Sci. Adv. Mater. 2013, 5, 1967–1973.

[19] F. Hof , S. Bosch , J. M. Englert , F. Hauke , A. Hirsch , Angew. Chem. Int. Ed. 2012, 51, 11727–11730; 10.1002/anie.201204791 23109244

[20a] W. A. Waters , J. Chem. Soc. 1942, 266–270;

[20b] T. Cohen , A. G. Dietz, Jr. , J. R. Miser , J. Org. Chem. 1977, 42, 2053–2058.

[21] A. C. Ferrari , J. Robertson , Phys. Rev. B 2000, 61, 14095.

[22a] F. N. Tebbe , R. L. Harlow , D. B. Chase , D. L. Thorn , G. C. Campbell , J. C. Calabrese , N. Herron , R. J. Young , E. Wasserman , Science 1992, 256, 822–825; 17756446 10.1126/science.256.5058.822

[22b] D. A. Dixon , N. Matsuzawa , T. Fukunaga , F. N. Tebbe , J. Phys. Chem. 1992, 96, 6107–6110.

[23] H. Telg , J. Maultzsch , S. Reich , F. Hennrich , C. Thomsen , Phys. Rev. Lett. 2004, 93, 177401. 15525124 10.1103/PhysRevLett.93.177401

[24] E. B. Starkey , Org. Synth. 1939, 19, 40.

