# Supplementary material for: Controlling the Degree of Functionalization: In‐Depth Quantification and Side‐Product Analysis of Diazonium Chemistry on SWCNTs
Source: Chemistry. 2019 Sep 5;25(55):12761–8. doi: 10.1002/chem.201902330 (PMC6790569; doi:10.1002/chem.201902330)
Supplement: Supplementary file 1 — Supplementary [file CHEM-25-12761-s001.pdf]

CHEMISTRY
A European Journal

Supporting Information

Controlling the Degree of Functionalization: In-Depth Quantification and Side-Product Analysis of Diazonium Chemistry on SWCNTs**

Milan Schirowski,[a, b] Frank Hauke,[a, b] and Andreas Hirsch*[a, b]

chem_201902330_sm_miscellaneous_information.zip
