# Supplementary figures and images for: Controlling the Degree of Functionalization: In‐Depth Quantification and Side‐Product Analysis of Diazonium Chemistry on SWCNTs
Source: Chemistry. 2019 Sep 5;25(55):12761–8. doi: 10.1002/chem.201902330 (PMC6790569; doi:10.1002/chem.201902330)

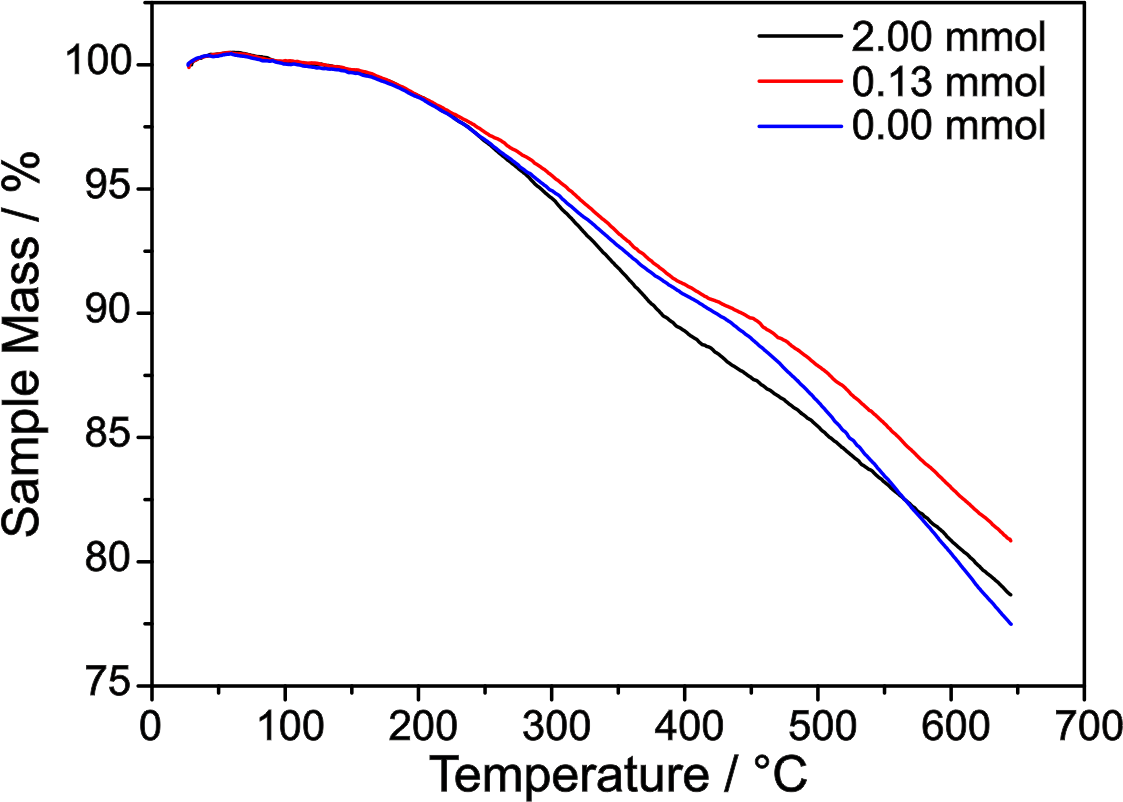

Supplement: Supplementary file 2 — Supplementary [file CHEM-25-12761-s002.zip › FigS1.png]

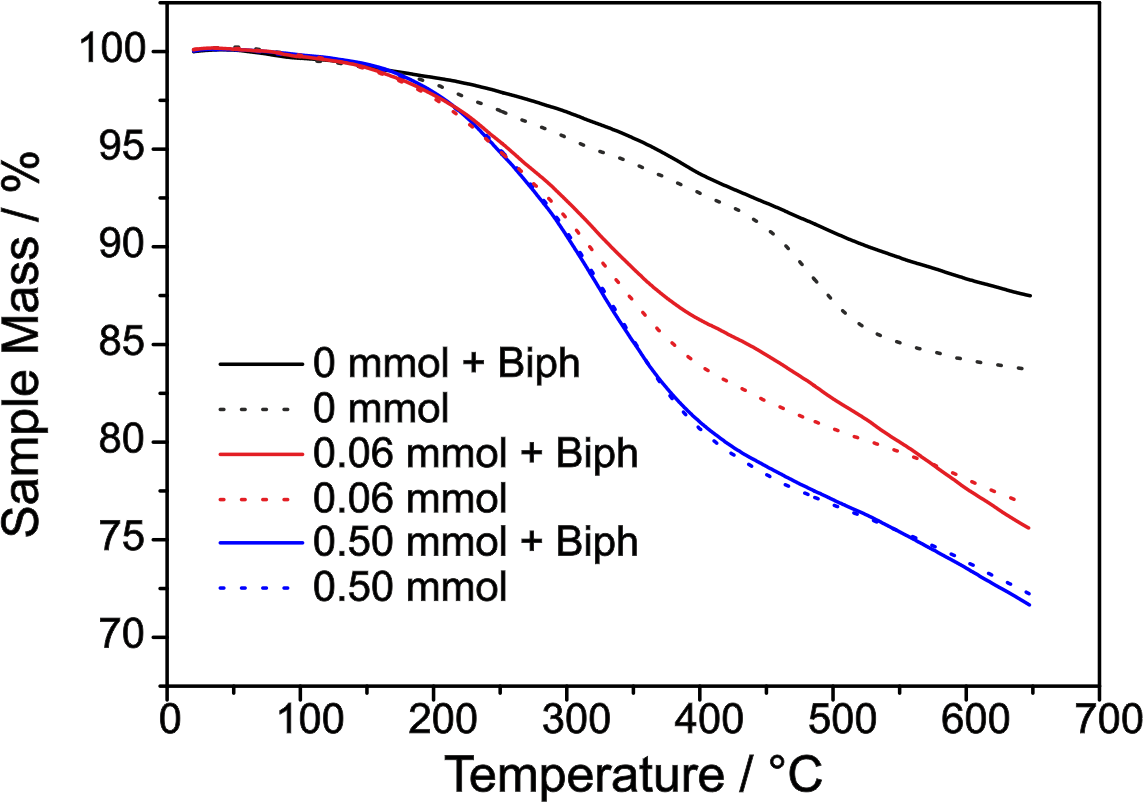

Supplement: Supplementary file 2 — Supplementary [file CHEM-25-12761-s002.zip › FigS2.png]

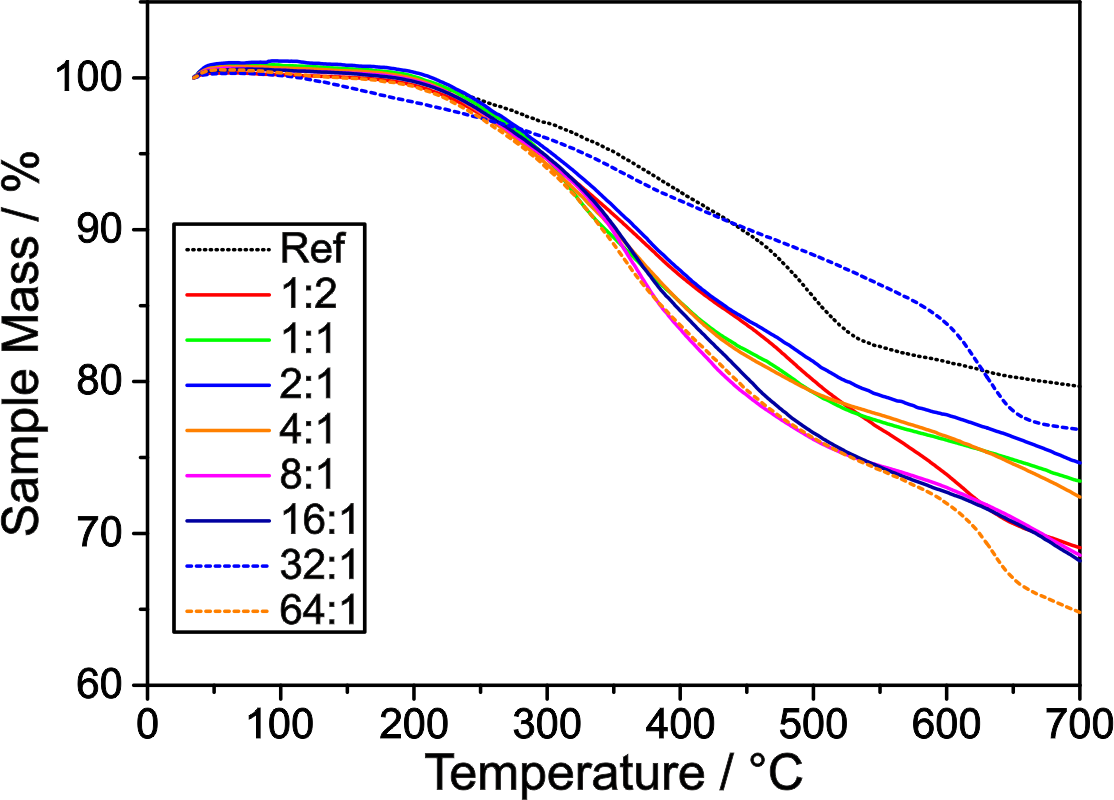

Supplement: Supplementary file 2 — Supplementary [file CHEM-25-12761-s002.zip › FigS3.png]

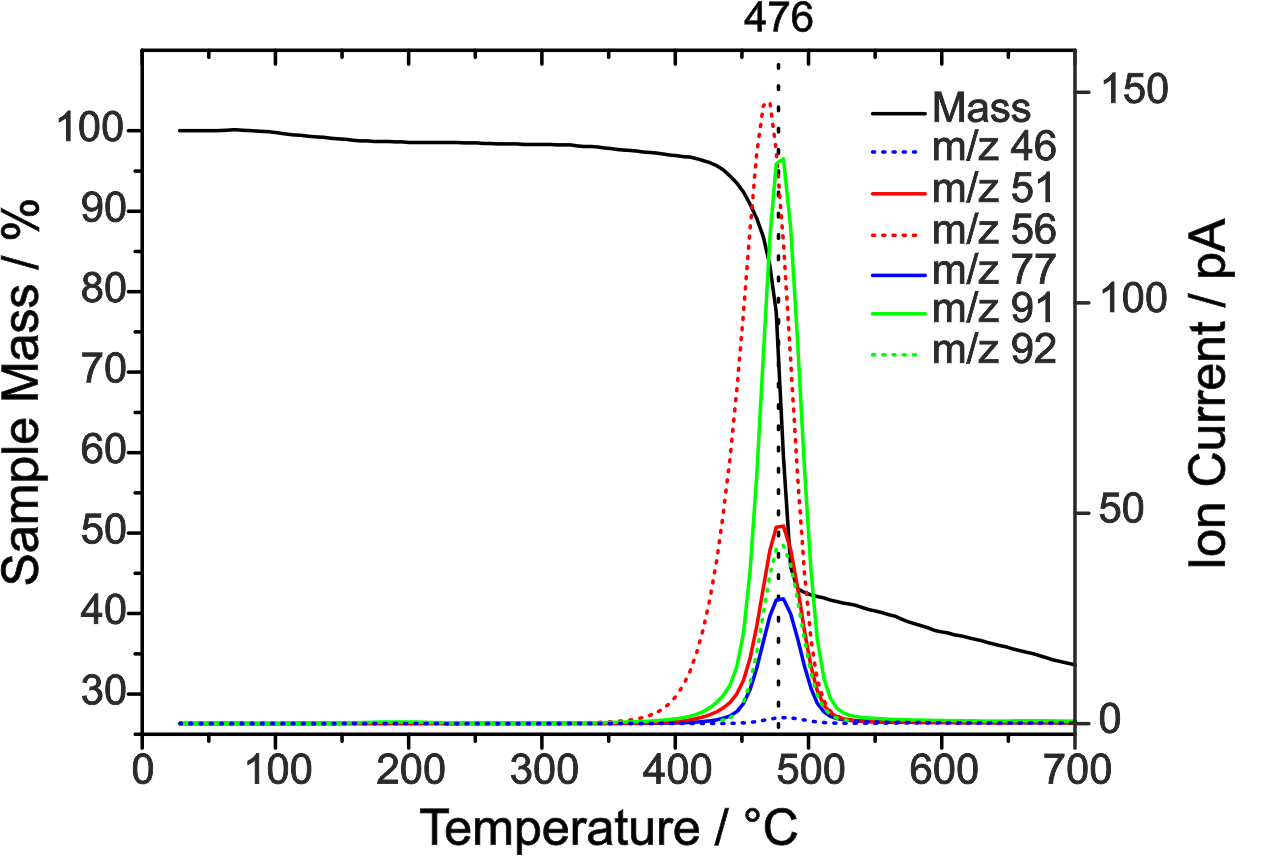

Supplement: Supplementary file 2 — Supplementary [file CHEM-25-12761-s002.zip › FigS4.png]

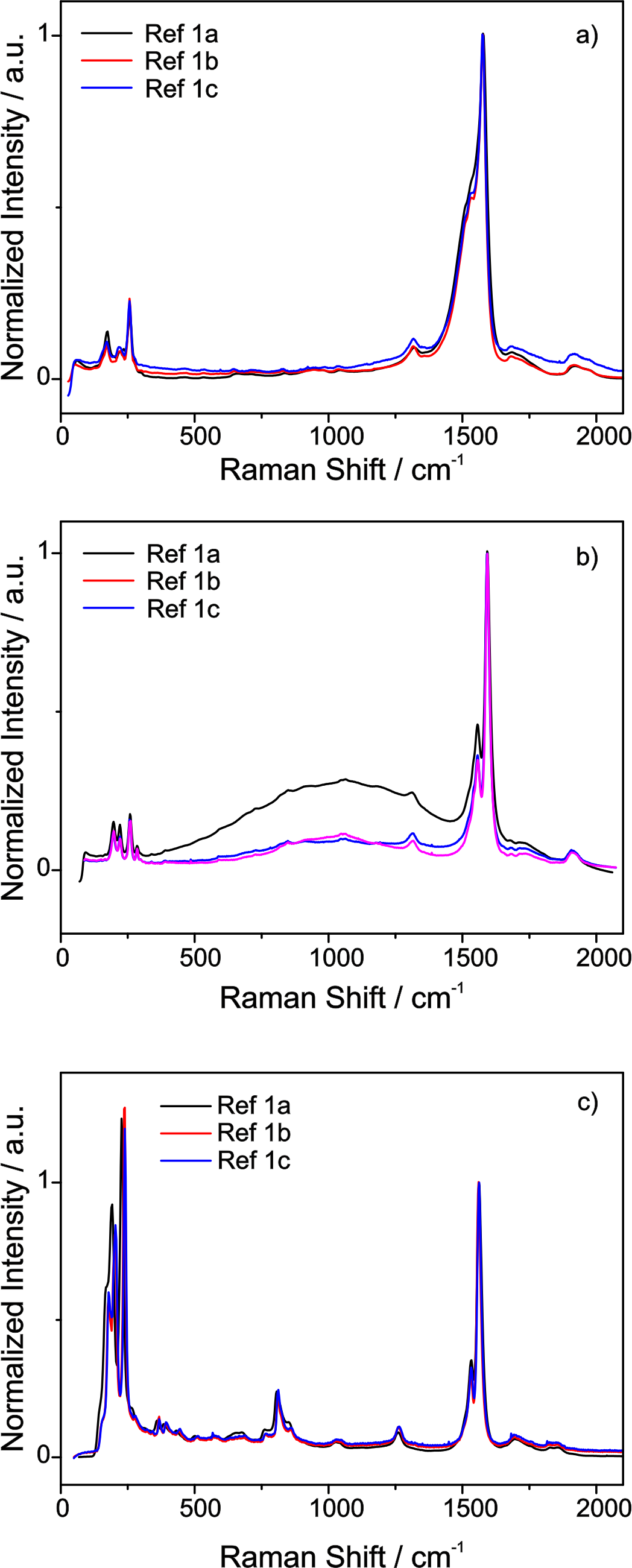

Supplement: Supplementary file 2 — Supplementary [file CHEM-25-12761-s002.zip › FigS5.png]
